# Orthodontic treatment during pregnancy, lactation, and postmenopausal period: a questionnaire development

**DOI:** 10.1590/1807-3107bor-2024.vol38.0013

**Published:** 2024-01-05

**Authors:** Juliana de Lourdes FERNANDES, Matheus França PERAZZO, Saul Martins PAIVA, Paulo Antônio MARTINS-JÚNIOR, Soraia MACARI

**Affiliations:** (a) Universidade Federal de Minas Gerais – UFMG, School of Dentistry, Department of Restorative Dentistry, Belo Horizonte, MG, Brazil.; (b) Universidade Federal de Minas Gerais – UFMG, School of Dentistry, Department of Oral Health of Children and Adolescents, Belo Horizonte, MG, Brazil.

**Keywords:** Pregnancy, Postmenopause, Lactation, Orthodontics, Validation Study, Surveys and Questionnaires

## Abstract

This study aimed to develop and validate a self-administered questionnaire in Brazilian Portuguese to verify the level of knowledge of orthodontists in the care of pregnant, lactating, and postmenopausal women, named “Considerations on Orthodontic Treatment during Pregnancy, Lactation, and Postmenopausal Periods.” The development and validation of the questionnaire consisted of the following steps: a) item generation; b) item reduction; c) questionnaire design; and d) validity and reliability tests in a cross-sectional study with 258 orthodontists working in the field from different Brazilian states. A total of 60 orthodontists participated in test-retest over a mean period of 45 days. The preliminary questionnaire consisted of a total of 60 questions. After item reduction, 40 questions were selected for the final version of the questionnaire, with eight questions about pregnant women; six about lactating women; 18 about postmenopausal women, and eight about general knowledge in dentistry. Each item had three response options in the Likert scale format. Face and content validity analysis, reliability assessment through internal consistency (Cronbach’s alpha and McDonald’s omega), and test-retest reliability through the intraclass correlation coefficient (ICC) and Spearman’s correlation coefficient were performed. Face and content validity indicated that the questionnaire was considered valid, objective, and easily understandable. The questionnaire had good internal consistency (Cronbach’s alpha = 0.77; McDonald’s omega = 0.78) and good test-retest reliability (ICC = 0.71; Spearman’s correlation coefficient = 0.51). The questionnaire was considered valid and reliable to assess the level of knowledge of orthodontists in the care of pregnant, lactating, and postmenopausal women.

## Introduction

Orthodontics is an expanding area and works extensively with other dental specialties to collaborate with the development or completion of complex oral rehabilitations. According to the American Association of Orthodontists, the number of adult patients undergoing orthodontic treatment increased by 14% between 2010 and 2012, of which 56% were women.^1^ These patients seek orthodontic treatment in both their fertile and postmenopausal periods.^2-4^

During different stages of life, such as during pregnancy, lactation, and postmenopausal period, women undergo major hormonal changes. During pregnancy and lactation, the woman’s body is subjected to a high calcium demand due to the development of the fetus and milk production, with an increase in the production of prolactin.^5-7^ Estrogen, which is the predominant hormone during a woman’s reproductive stage, abruptly decreases its concentration during the postmenopausal period, which can lead to osteoporosis.^8^ Several studies report the effect of these female hormones on maxillary bone remodeling and their influence on the craniofacial complex and on orthodontic tooth movement.^4,9-11^

Despite the existing knowledge in the literature about bone and orthodontic tooth movement alterations during pregnancy, lactation, and postmenopausal periods, there are no reports on how orthodontists conduct treatments in these groups of patients. It is of utmost importance that orthodontists be aware of the effects of hormones during the different stages of a woman’s life so that their clinical approach may be appropriate according to the demand of the female body, favoring the success of orthodontic treatment.^4,12^ Therefore, we identified the need to develop and validate a relevant assessment instrument to improve and adapt the care provided by orthodontists to women during these periods of their lives.

This study aimed to develop and validate a self-administered questionnaire in Brazilian Portuguese to verify the level of knowledge of orthodontists in the care of pregnant, lactating, and postmenopausal women, named “Considerations on Orthodontic Treatment during Pregnancy, Lactation, and Postmenopausal Periods.” This study hypothesizes that the questionnaire is valid and reliable to verify the level of knowledge of orthodontists in the care of pregnant, lactating, and postmenopausal women.

## Methodology

### Ethical considerations

According to the ethical principles for medical research on human beings – Declaration of Helsinki – the research was approved by the Research Ethics Committee (REC) of the Federal University of Minas Gerais (UFMG) (CAAE: 31864520.9.0000.5149). All the participants of this research have consented to their participation through an informed consent form (ICF).

### Instrument development

**Figure 1:**
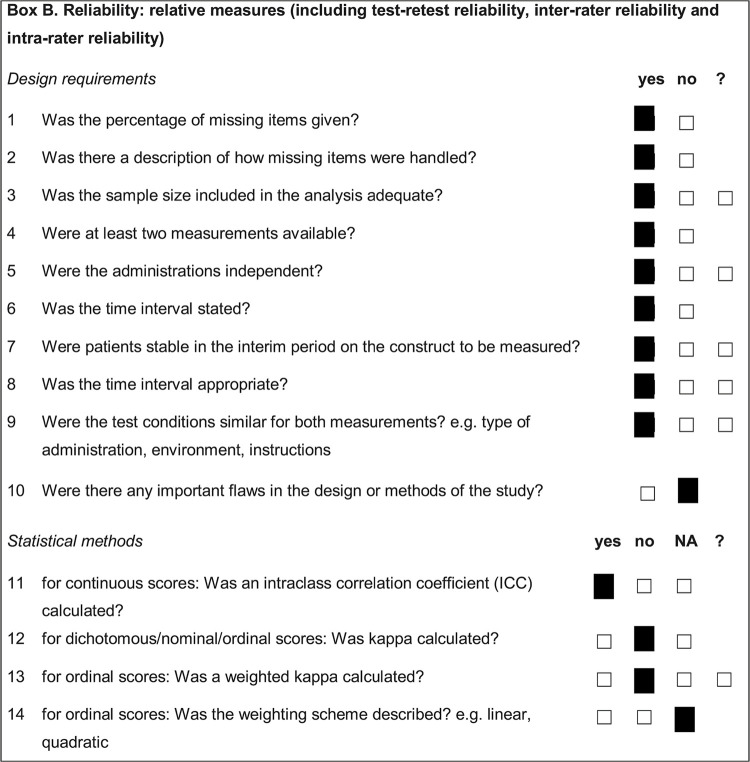
COSMIN (COnsensus-based Standards for selection of health Measurement INstruments) Box B (reliability) checklist.

**Figure 2:**
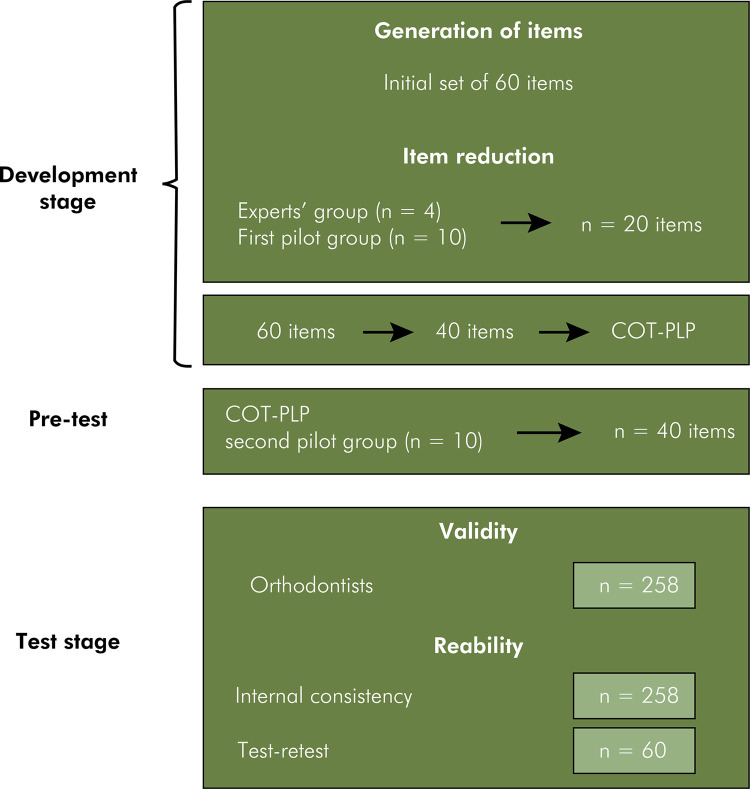
Flowchart describing the development and validation of the “Considerations on Orthodontic Treatment during Pregnancy, Lactation, and Postmenopausal Periods” (COT-PLP) instrument.

The instrument was developed according to validated protocols for questionnaire development and validation, and the COSMIN (COnsensus-based Standards for selection of health Measurement INstruments) Box B (reliability) checklist was used to assess the methodological quality of the study design (Figure 1),^13-16^ consisting of the following steps: a) item generation; b) item reduction; c) questionnaire design; and d) survey validity and reliability tests (Figure 2).

### Item generation

For item generation, a focus group of experts composed of two PhD professors of pediatric dentistry experienced in the development of questionnaire in the health area, a PhD professor of orthodontics specialized in bone remodeling, mainly in female models, all of them affiliated with the Faculty of Dentistry of the Federal University of Minas Gerais (FAO-UFMG) and, a physician specialized in gynecology and obstetrics, working in a private practice. The expertise group held 19 meetings for developing and adapting the questionnaire items. To address the themes in each section, the expertise group considered the following criteria: the most relevant scientific literature related to the orthodontic treatment during pregnancy, lactation, and postmenopausal periods, the different specific knowledge of each expert, and the physician’s experience in clinical practice. The sociodemographic data of the orthodontists were also included in the questionnaire, consisting of: sex, age, state of origin, time of specialization experience, and whether the title of orthodontist was obtained from a public or private faculty or class entities. Finally, the following thematic sections were considered: sociodemographic data, orthodontic treatment in pregnant, lactating, and postmenopausal women, and general clinical issues.

Based on the literature review and on the expertise meetings, a preliminary questionnaire with 60 questions and a five-item response model based on the Likert scale was created and structured as follows: 11 items on knowledge about the care of pregnant women, 11 items on knowledge about the care of lactating women, 27 items on knowledge about the care of postmenopausal women, and 11 questions about general knowledge in dentistry. After the construction and analysis of this first version of the questionnaire, the group of experts decided to add, below each question, a question about the level of relevance of that item: high relevance, medium relevance, or low relevance. At the end of the questionnaire, the respondents could make suggestions, thus giving rise to a second version of the preliminary questionnaire.

### Item reduction

Two pilot groups were set up (n = 10 each) for item reduction and questionnaire design. The 20 specialists in the field of orthodontics were selected randomly from different regions of Brazil through professional associations. The first pilot group composed of 10 specialists in orthodontics was invited to remotely answer the preliminary questionnaire in its second version, using Google Forms. The goal was to verify the degree of understanding of orthodontists regarding the questions asked, as well as to assess the level of relevance of each item and welcome possible suggestions for improvement. Based on the analysis of the responses given by the pilot group mentioned above, those items that received two or more low scores in terms of their level of relevance were excluded (Table 1), leading to a reduction in the number of items in the questionnaire from 60 to 40. A question was added below each item, asking whether the item above was clear. Also, if the answer to the question was “no,” a justification was requested to allow making a subsequent change to that item. After the first pilot test, the response model changed from five to three items: agree, neither agree nor disagree, and disagree.

**Table 1:** Results of the pilot test regarding the level of relevance of the items.

Consensus on item reduction	Justification for exclusion
1. Orthodontists should always be aware of the systemic condition and medical history of their patients.	Low relevance
2. Orthodontists should always take notes of the medication used by their patients.	Low relevance
6. Estrogen is an important regulator of bone metabolism not only in women, but also in men.	Low relevance
9. Orthodontic tooth movement is performed by remodeling the alveolar bone in response to the mechanical force caused by the orthodontic appliance.	Low relevance
10. Local and systemic factors also influence bone remodeling during orthodontic treatment.	Low relevance
17. Gingivitis gravidarum is a common oral disorder in pregnancy.	Low relevance
18. Pregnant patients should undergo prophylactic procedures more often during pregnancy, especially if they are undergoing orthodontic treatment.	Low relevance
24. Orthodontists can perform orthodontic treatment on lactating patients.	Low relevance
25. It is important that lactating patients be monitored by both their orthodontist and their physician.	Low relevance
28. Lactating patients should undergo prophylactic procedures more often, especially if they are undergoing orthodontic treatment.	Low relevance
30. After the lactation period, a woman may have osteoporosis, which occurs during a transitory period in which the woman has more fragile bones.	Low relevance
34. Orthodontists may request orthodontic documentation from postmenopausal patients.	Low relevance
35. Orthodontists can perform orthodontic treatment on patients who have already entered menopause.	Low relevance
36. Orthodontists can perform orthodontic treatment on postmenopausal patients.	Low relevance
38. Postmenopausal women are more likely to acquire systemic diseases such as diabetes and/or hypertension.	Low relevance
39. It is important that postmenopausal patients be monitored by both their orthodontist and their physician.	Low relevance
48. In case of diagnosis of a patient suspected of having osteoporosis, the best approach is to refer the patient to a physician.	Low relevance
49. Height loss and bone fractures in the vertebrae and/or femur may be signs of osteoporosis.	Low relevance
52. Bisphosphonates are the drugs of choice for the treatment of osteoporosis.	Low relevance
56. Estrogen is the predominant hormone during a woman’s reproductive phase, with its abrupt breakdown during the postmenopausal period.	Low relevance
60. Answering this survey makes you think thoroughly about the care, planning, and conduct of orthodontic treatment on pregnant, lactating, and postmenopausal women.	Low relevance

Questions that received two or more low relevance ratings were excluded.

### Questionnaire design

The second pilot group, composed of other 10 specialists in orthodontics, was invited to participate in the study, also remotely, to assess the objectivity of the questions proposed in the second version of the pilot questionnaire, using Google Forms. After analysis of the answers of the second pilot group by the group of experts, general revisions were made to the statement of each item, misspellings were corrected, and some questions were rephrased, thus establishing the final version of the questionnaire in a self-administered format with 40 items: eight items on knowledge about the care of pregnant women, such as radiographic exams during pregnancy, communication between orthodontists and gynecologists, calcium and vitamin supplementation, and periodicity of prophylactic procedures; six items on the care provided to lactating women regarding hormone replacement, calcium requirements, bone remodeling, and tooth movement; 18 items on knowledge about care in postmenopausal women, such as osteoporosis, physical activity, bone density, hormone replacement, diagnostic tests, and use of bisphosphonates; and eight items on general knowledge in dentistry, such as anamnesis, action of calcium and vitamin D, bone remodeling, and care protocol. The Likert scale was used in the response model, with three response options: “agree,” “neither agree nor disagree,” and “disagree.” The answers were evaluated dichotomously, where 1 indicated a correct answer and 0 indicated a wrong answer. The total sum varied from 0 to 40, and the sums of the thematic sections were as follows: pregnant women (0–8), lactating women (0–6), postmenopausal women (0–18), and general knowledge (0–8). The “neither agree nor disagree” answer contained three items, the “disagree” answer had 11 items, and the “agree” answer included the remainder of the items. The higher the score obtained, the higher the level of knowledge of orthodontists about the subject.

The theoretical single domain of the questionnaire was qualitatively assessed through face and content validity. During face validity, it was verified whether the items were able to assess what they were intended to. Content validity verified whether the questionnaire contained questions that covered all aspects of the construct to be assessed.^14^ Questionnaire validity is defined as the degree to which a measurement quantifies what it intends to measure; in this case, based on the literature and experts. Face and content validity is an essential step for the development of new tests in order to assess the degree of representativeness of the items in the questionnaire standardization process. In our study, face and content validity was assessed through the application of the preliminary version of the instrument in the pilot and expert groups. In the first step, for face validity, both the suggestions made by both pilot groups of orthodontists, as well as their answers to and suggestions for each question of the questionnaire were analyzed, generating substantial and important changes in the construct. Subsequently, for content validity, a more meticulous and detailed analysis of the question, based on the scientific literature and grammatical rules, was performed by the experts, thus establishing the 40 questions. At this stage, it was possible to verify the degree of understanding of the orthodontists in relation to the formulated items, as well as to assess the level of relevance of each item.

### Instrument validity and reliability

For the validity and reliability tests, the number of participants (N) was defined according to the sample calculation method,^14,16,17^ in which N should be 5 to 7 times greater than the number of items in the survey (40 items x 5 = 200), including at least 100 participants. Therefore, the survey was applied to a sample group consisting of 258 orthodontists working in the field from different Brazilian states, and who had not participated in the previous pilot groups. The participants were randomly selected from different regions of Brazil through professional associations. Dentists specialized in orthodontics, regardless of age, sex, or training period, were included. Those specialists who were not working in the field were excluded from the study. Professionals were contacted by email via class entities. The email messages contained the link to the questionnaire, information about the study, the study number in the REC, and the ICF.

The questionnaire’s reliability was assessed through internal consistency and test-retest. To verify internal consistency, Cronbach’s alpha and McDonald’s omega were used. After a 4-week interval, 60 orthodontists (approximately 20% of the participants)^18^ were invited to participate in the test-retest analysis with a mean (range) of 45 (5–69) days from the initial assessments. Test-retest reliability was assessed using Spearman’s correlation and the intraclass correlation coefficient (ICC).

The statistical tests run in this study were performed using the Statistical Package for Social Sciences (SPSS for Windows, version 26.0, SPSS Inc. Chicago, USA). The results were expressed as average ± standard deviation (SD).

## Results

The validity and reliability of the instrument were assessed in a sample of 258 Brazilian orthodontists. The number of orthodontists was not proportionally distributed by region of the country (Table 2). Table 3 presents the absolute and relative values related to the characteristics of the participants. The mean age of participants was 44.04 (standard deviation of ±11.0) years, minimum of 30 years and maximum of 77 years, and 60.1% of the sample consisted of women. Table 4 shows the average, central tendency, and observed ranges for the total score of the questionnaire, as well as for each group of questions.

**Table 2:** Distribution of the number of orthodontists by Brazilian region

States	Number	Percentage
Minas Gerais (MG)	60	23.3%
Mato Grosso do Sul (MS)	2	0.8%
Paraíba (PB)	1	0.4%
Paraná (PR)	19	7.4%
Rio de Janeiro (RJ)	37	14.3%
Rio Grande do Norte (RN)	1	0.4%
São Paulo (SP)	39	15.1%
Mato Grosso (MT)	1	0.4%
Santa Catarina (SC)	16	6.2%
Paraná (PR)	2	0.8%
Rio Grande do Sul (RS)	16	6.2%
Goiás (GO)	16	6.2%
Alagoas (AL)	5	1.9%
Espírito Santo (ES)	3	1.2%
Pará (PA)	3	1.2%
Sergipe (SE)	4	1.6%
Ceará (CE)	6	2.3%
Bahia (BA)	7	2.7%
Piauí (PI)	4	1.6%
Pernambuco (PE)	8	3,10%
Amazonas (AM)	1	0.4%
Sergipe (SE)	1	0.4%
Distrito Federal (DF)	3	1.2%
Maranhão (MA)	1	0.4%
Tocantins (TO)	1	0.4%
Roraima (RR)	1	0.4%
Total	258	100

**Table 3:** Descriptive analysis of sociodemographic variables related to orthodontists (n = 258)

Variables	n (%)
Sociodemographic variables	
Sex	
Female	155 (60.1)
Male	103 (39.9)
Age	258
Where the specialist obtained his/her degree from	
Private institution	90 (34.9)
Public institution	99 (38.4)
Class entities	69 (26.7)
Time to graduation	
Up to 10 years	104 (40.3)
More than 10 years	154 (59.7)

**Table 4:** Mean, standard deviation (SD), median, and variation of COT-PLP scores (n = 258)

COT-PLP	Mean (SD)	Median	Variation
COT-PLP total score (0–50)	23.45	24	10–34
Pregnant women section (0–7)	4.00 (1.33)	4	1–7
Lactating women section (0–10)	2.80 (1.01)	3	0–5
Postmenopausal women section (0–21)	10.72 (3.14)	11	2–17
General knowledge section (0–12)	5.92 (1.55)	6	1–8

COT-PLP: considerations on orthodontic treatment during pregnancy, lactation and postmenopause; SD: standard deviation.

During the item reduction process, based on the analysis of the responses given by the first pilot group, 21 items were excluded from the instrument (Table 1), in addition to the generation of a new item - “A special orthodontic care protocol is required for pregnant, lactating, and postmenopausal women” –, which resulted in a 40-item instrument, consisting of eight items on knowledge about care for pregnant women, six items on knowledge about care for lactating women, 18 items on knowledge about care for postmenopausal women, and eight items on general knowledge about dentistry. The participants took approximately 15 minutes to answer the questionnaire. In addition to these important changes, the response model changed from five to three items, consisting of “agree,” “neither agree nor disagree,” and “disagree” options. Based on the analysis of the responses obtained from the second pilot group, three items were reformulated in the postmenopausal section, as well as two items in the general knowledge section, resulting in the final version of the instrument. Additionally, the order of the sections was changed to give a greater focus on the main objective of the study, establishing the following thematic sections regarding the assessment of the level of knowledge of orthodontists about care: in a) pregnant women – 8 items; b) lactating women – 6 items; c) postmenopausal women – 18 items; and d) general knowledge on dentistry – 8 items.

Through qualitative analysis of face validity (by orthodontists) and content validity (by the group of experts), the questionnaire was considered valid, objective, and easy to understand. The questions were clear and the content and answer options were appropriate for what was being evaluated. Table 5 presents the construct plausibility after face and content validity and the pertinent justifications and references of each item of the construct.

**Table 5:** Construct items and answers after face and content validation with justification for the question and references used.

Sections/Items/Answers	Justification	References
Pregnant women		
1.The orthodontist may request radiographic examinations of pregnant patients.	In non-emergency situations, requests for radiographies between the 10th and 17th week of pregnancy should be avoided, as this is the period of greatest sensitivity of the embryo’s central nervous system.	TOPPENBERG et al. (1999) Safety of radiographic imaging during pregnancy.Am Fam Physician.
Answer: I do not agree nor disagree		
2. Even with the orthodontic documentation ready, the patient cannot begin orthodontic treatment if they are in the first trimester of pregnancy.	Orthodontic treatment can be performed during pregnancy, once both patient and orthodontist are aware of the importance of maintaining oral hygiene as well as prophylactic and treatment strategies for managing gingival health during pregnancy.	SACHAN et al. (2013) Considerations for the orthodontic treatment during pregnancy. https://doi.org/10.4103/2321-3825.123321
Answer: Disagree		
3. During orthodontic treatment, communication between the orthodontist and the pregnant woman’s obstetrician/gynecologist is mandatory.	Communication between gynaecologists and orthodontists is crucial and relevant to the care of pregnant women during the prenatal period.	GOVINDASAMY et al. (2018) Knowledge, awareness, and practice among gynecologists, medical practitioners and dentists in Madurai regarding association between periodontitis and pregnancy outcomes. https://doi.org/10.4103/jisp.jisp_164_18; PATIL et al. (2013) Oral Health Coalition: Knowledge, Attitude, Practice Behaviours among Gynaecologists and Dental Practitioners.
Answer: Disagree		
4. Women in the third trimester of pregnancy who are not intaking the adequate amount of calcium and who report constant pain in the lower back are able to put on orthodontic appliances.	There is an increasing demand for calcium in the pregnant women’s body for fetal development and this process interferes with calcium homeostasis and bone remodelling.	CROSS et al. (1995) Calcium homeostasis and bone metabolism during pregnancy, lactation, and postweaning: a longitudinal study. https://doi.org/10.1093/ajcn/61.3.514; KOVACS et al. (2005) Calcium and bone metabolism during pregnancy and lactation. J Mammary Gland Biol Neoplasia. https://doi.org/10.1016/j.ecl.2011.08.002
Answer: Disagree		
5. During orthodontic treatment in pregnant women, the orthodontist should check if the patients are supplementing calcium.	Calcium insufficiency and deficiency have not been sufficiently studied in humans to establish what levels of supplementation are necessary or optimal, but adequate pregnant women’s vitamin D supplementation should be maintained during pregnancy.	HOLLIS et al. (2004) Assessment of dietary vitamin D requirements during pregnancy and lactation. https://doi.org/10.1093/ajcn/79.5.717 KALKWARF et al. (1997) The effect of calcium supplementation on bone density during lactation and after weaning. https://doi.org/10.1056/NEJM199708213370803https://doi.org/
Answer: Agree		
6. Pregnant and undergoing orthodontic treatment patients should perform prophylactic procedures more frequently to prevent the occurrence of gingivitis gravidarum.	Studies have shown that orthodontists should be more aware of the oral health of their patients, with an emphasis on guidance, maintenance of oral hygiene and prophylactic strategies for maintaining gingival health, since orthodontic appliances can act as a source of plaque retention and aggravate inflammatory reactions.	BERLIN-BRONER et al. (2012) Awareness of orthodontists regarding oral hygiene performance during active orthodontic treatment. Eur Arch Paediatr Dent.; SACHAN et al. (2013) Considerations for the orthodontic treatment during pregnancy. https://doi.org/10.4103/2321-3825.123321
Answer: Agree		
7. The demand for calcium remains unchanged in the pregnant woman’s body.	There is an increasing demand for calcium during all trimesters of pregnancy.	CROSS et al. (1995) Calcium homeostasis and bone metabolism during pregnancy, lactation, and postweaning: a longitudinal study. https://doi.org/10.1093/ajcn/61.3.514; KOVACS et al. (2005) Maternal Mineral and Bone Metabolism During Pregnancy, Lactation, and Post-Weaning Recovery. https://doi.org/10.1152/physrev.00027.2015
Answer: Disagree		
8. After the gestation period, women may have transient gestational osteoporosis.	An osteoporosis in the hip associated with pregnancy may occur, and this event is rare and transitory.	KOVACS et al. (2005) Maternal Mineral and Bone Metabolism During Pregnancy, Lactation, and Post-Weaning Recovery. https://doi.org/10.1152/physrev.00027.2015
Answer: Agree		
Lactating women		
1. The orthodontist may perform orthodontic treatment in lactating patients.	The orthodontist should be aware that mechanical force-induced bone remodelling is increased during the lactation period.	MACARI et al. (2018) Lactation induces increases in the RANK/RANKL/OPG system in maxillary bone. https://doi.org/10.1016/j.bone.2018.01.032
Answer: Agree		
2. During orthodontic treatment in lactating women, the orthodontist should check if the patients are supplementing calcium.	Supplementation of lactating patients is believed to have the following purposes: to increase the patient’s vitamin D nutritional status and thereby improve vitamin D nutrition.	CROSS et al. (1995) Changes in bone mineral density and markers of bone remodeling during lactation and postweaning in women consuming high amounts of calcium. https://doi.org/10.1002/jbmr.5650100907; KALKWARF et al. (1997) The effect of calcium supplementation on bone density during lactation and after weaning. https://doi.org/10.1056/NEJM199708213370803
Answer: Agree		
3. During the lactation period, women have a high demand for calcium, for both maintaining bone mineral homeostasis and milk production.	The demand for bone mineral from women’s skeleton is considered a normal outcome of lactation.	CROSS et al. (1995) Calcium homeostasis and bone metabolism during pregnancy, lactation, and postweaning: a longitudinal study. https://doi.org/10.1093/ajcn/61.3.514; KOVACS et al. (2005) Maternal Mineral and Bone Metabolism During Pregnancy, Lactation, and Post-Weaning Recovery. https://doi.org/10.1152/physrev.00027.2015
Answer: Agree		
4. Lactating patients should undergo prophylactic procedures more often, especially if they are undergoing orthodontic treatment.	Lactating patients should undergo prophylactic procedures more often, as orthodontic appliances can act as a source of plaque retention and aggravate inflammatory reactions.	BERLIN-BRONER et al. (2012) Awareness of orthodontists regarding oral hygiene performance during active orthodontic treatment. Eur Arch Paediatr Dent.; SACHAN et al. (2013) Considerations for the orthodontic treatment during pregnancy. https://doi.org/10.4103/2321-3825.123321
Answer: I do not agree nor disagree		
5. Lactating women lose 1 to 3% of their bone mineral content per month.	This loss is an expected consequence of the lactation process.	CROSS et al. (1995) Changes in bone mineral density and markers of bone remodeling during lactation and postweaning in women consuming high amounts of calcium. https://doi.org/10.1002/jbmr.5650100907
Answer: I do not agree nor disagree		
6. During lactation, the acceleration of orthodontic tooth movement may occur associated with transient bone loss, which is characteristic of this stage.	This event occurs due to the increased differentiation of osteoclasts and osteoblasts during lactation.	MACARI et al. (2018) ST2 regulates bone loss in a site-dependent and estrogen-dependent manner. https://doi.org/10.1002/jcb.27080
Answer: Agree		
Postmenopausal women		
1. Postmenopausal women are more likely to develop osteoporosis.	Osteoporosis is a prevalent disease in female patients and is associated with the postmenopausal period.	TARITY et al. (2013) Mortality in centenarians with hip fractures. https://doi.org/10.3928/01477447-20130222-15
Answer: Agree		
2. A woman who performs physical activities and eats properly loses bone density after 40 years of age.	Adequate nutrition associated with physical activity has the ability to maintain bone homeostasis by increasing osteoblastic activity, bone mineral density and trabecular bone volume in the alveolar bone.	OLIVEIRA et al. (2020) Bovine Milk Extracellular Vesicles Are Osteoprotective by Increasing Osteocyte Numbers and Targeting RANKL/OPG System in Experimental Models of Bone Loss. https://doi.org/10.3389/fbioe.2020.00891; PASQUALINI et al. (2019) Effects of a 3-month weight-bearing and resistance exercise training on circulating osteogenic cells and bone formation markers in postmenopausal women with low bone mass. https://doi.org/10.1007/s00198-019-04908-9;
Answer: Disagree		
3. During orthodontic treatment in postmenopausal women, the orthodontist should check if the patients are undergoing hormonal replacement.	The effect of female hormones, such as prolactin and estrogen, on bone remodelling and their influence on the craniofacial complex.	CLÉMENT-LACROIX et al. (1999) Osteoblasts are a new target for prolactin: analysis of bone formation in prolactin receptor knockout mice. https://doi.org/10.1210/endo.140.1.6436; MACARI et al. (2016) Osteoprotective effects of estrogen in the maxillary bone depend on ERα. https://doi.org/10.1177/0022034516633154
Answer: Agree		
4. Postmenopausal estrogen-deficient patients show reduced risk of root resorption during orthodontic treatment.	Estrogen deficiency accelerates orthodontic movement and may interfere with clinical outcomes.	AMARO et al. (2020) Estrogen protects dental roots from orthodontic-induced inflammatory resorption.
Answer: Disagree		
5. Multiple tooth loss and low mandibular bone density may be intraoral signs in women already presenting osteoporosis.	The dentist can be the first health professional to early diagnose osteoporosis, a decrease in alveolar bone even with proper hygiene may indicate the need for further systemic screening of bone mineral density.	KINALSKI et al. (2019) The accuracy of panoramic radiography as a screening of bone mineral density in women: a systematic review. https://doi.org/10.1259/dmfr.20190149; LEITE et al. (2015) Systematic review with hierarchical clustering analysis for the fractal dimension in assessment of skeletal bone mineral density using dental radiographs. https://doi.org/10.1007/s11282-014-0188-y; STEWART et al. (2012) Building osteoporosis prevention into dental practice.
Answer: Agree		
6. Osteopenia can be understood as the stage that precedes osteoporosis, occurring when bone resorption is greater than bone neoformation.	Many women experience a long period of osteopenia before being considered as being affected by osteoporosis.	PASCO et al. (2006) The population burden of fractures originates in women with osteopenia, not osteoporosis. https://doi.org/10.1007/s00198-006-0135-9
Answer: Agree		
7. Early menopause, such as having the ovaries removed before the age of 50, reduces the chances of developing osteoporosis.	Hormone replacement, proper nutrition and physical activity, among other factors, can reduce the likelihood of developing osteoporosis.	PASQUALINI et al. (2019) Effects of a 3-month weight-bearing and resistance exercise training on circulating osteogenic cells and bone formation markers in postmenopausal women with low bone mass. https://doi.org/10.1007/s00198-019-04908-9
Answer: Disagree		
8. Osteoporosis is caused by decreased calcium absorption.	The abrupt drop in female hormones can lead to osteoporosis.	WINDAHL et al. (2002) Elucidation of estrogen receptor function in bone with the use of mouse models.
Answer: Disagree		
9. Accelerated loss of bone mass is observed in osteoporosis.	Postmenopausal women with osteoporosis lose 1 to 3% of their bone mineral content per year.	CROSS et al. (1995) Changes in bone mineral density and markers of bone remodeling during lactation and postweaning in women consuming high amounts of calcium. https://doi.org/10.1002/jbmr.5650100907
Answer: Agree		
10. Dental radiographs are ineffective in the diagnose patients with suspect of osteoporosis.	The most effective method for diagnosing osteoporosis is bone densitometry.	HAILEY et al. (1996) The effectiveness of bone density measurement and associated treatments for prevention of fractures: an international collaborative review.
Answer: Disagree		
11. At present, the most acceptable method for diagnosing osteoporosis is bone densitometry (DEXA).	Bone densitometry has diagnostic and prognostic capability.	HAILEY et al. (1996) The effectiveness of bone density measurement and associated treatments for prevention of fractures: an international collaborative review.
Answer: Agree		
12. Postmenopausal women with osteoporosis who do not perform physical activities, do not eat properly, and do not undergo hormone replacement therapy can lose bone mineral content.	Accelerated loss of bone mass is observed in women with osteoporosis and without proper management of calcium intake and hormone replacement.	FESKANICH et al. (2003) Calcium, vitamin D, milk consumption, and hip fractures: a prospective study among postmenopausal women. https://doi.org/10.1093/ajcn/77.2.504
Answer: Agree		
13. Bisphosphonates, widely used in the treatment of osteoporosis, are associated with the occurrence of osteonecrosis of the jaws.	Bisphosphonates are the first-line drugs used to treat women who develop postmenopausal osteoporosis, but there is an increased incidence of osteonecrosis of the jaw as a side effect.	HELLSTEIN et al. (2011) Managing the care of patients receiving antiresorptive therapy for prevention and treatment of osteoporosis: executive summary of recommendations from the American Dental Association Council on Scientific Affairs. https://doi.org/10.14219/jada.archive.2011.0108
Answer: Agree		
14. Bisphosphonates do not interfere with neither the planning nor the outcome of orthodontic treatment.	The administration of bisphosphonates may be associated with increased treatment time and moderate changes such as remodelling in tooth roots and surrounding tissues in orthodontic patients.	SIDIROPOULOU-CHATZIGIANNIS et al. (2007) The effect of osteoporosis on periodontal status, alveolar bone and orthodontic tooth movement. A literature review.
Answer: Disagree		
15. Hormone replacement after menopause reduces the likelihood of bone fracture.	Hormone replacement is important for the maintenance bone structure.	MACARI et al. (2015) Oestrogen regulates bone resorption and cytokine production in the maxillae of female mice. https://doi.org/10.1016/j.archoralbio.2014.11.010
Answer: Agree		
16. The sharp reduction of estrogen levels in postmenopausal women is directly associated to bone loss and development of osteoporosis.	Hormonal deficiency is directly related to bone loss and the development of osteoporosis.	MACARI et al. (2018) ST2 regulates bone loss in a site-dependent and estrogen-dependent manner. https://doi.org/10.1002/jcb.27080
Answer: Agree		
17. Estrogen deficiency during the post-menopause period accelerates orthodontic tooth movement.	Estrogen deficiency accelerates orthodontic movement, interfering with the treatment approach and clinical outcomes.	MACARI et al. (2016) Osteoprotective effects of estrogen in the maxillary bone depend on ERα. https://doi.org/10.1177/0022034516633154; MACARI et al. (2018) Lactation induces increases in the RANK/RANKL/OPG system in maxillary bone. https://doi.org/10.1016/j.bone.2018.01.032
Answer: I do not agree nor disagree		
18. Physical activity and diet are not important factors in maintaining bone health in postmenopausal women.	Appropriate nutrition associated with physical activity has the ability to maintain bone homeostasis.	OLIVEIRA et al. (2020) Bovine Milk Extracellular Vesicles Are Osteoprotective by Increasing Osteocyte Numbers and Targeting RANKL/OPG System in Experimental Models of Bone Loss. https://doi.org/10.3389/fbioe.2020.00891; PASQUALINI et al. (2019) Effects of a 3-month weight-bearing and resistance exercise training on circulating osteogenic cells and bone formation markers in postmenopausal women with low bone mass. https://doi.org/10.1007/s00198-019-04908-9
Answer: Disagree		
General knowledge		
1. Orthodontists should always take notes of the medications used by their patients.	The orthodontist must always be aware of the systemic condition and medical history of their patients, as well as record all medications used by their patients throughout the treatment.	PATIL et al. (2013) Oral Health Coalition: Knowledge, Attitude, Practice Behaviours among Gynaecologists and Dental Practitioners.
Answer: Agree		
2. Calcium and vitamin D have the effect of preserving bone mass in women.	Both calcium and vitamin D take part in maintaining bone density.	KALKWARF et al. (1997); KOVACS et al. (2005)
Answer: Agree		
3. Some hormones, such as parathyroid hormone, estrogen, glucocorticoids and vitamin D3, play an insignificant role in the bone remodelling process.	Female hormones play a key role in the bone remodelling process.	MACARI et al. (2016) Osteoprotective effects of estrogen in the maxillary bone depend on ERα. https://doi.org/10.1177/0022034516633154; MACARI et al. (2018) ST2 regulates bone loss in a site-dependent and estrogen-dependent manner. https://doi.org/10.1002/jcb.27080
Answer: Disagree		
4. Cytokines and growth factors also act in the control of bone cell activity.	Local factors such as cytokines and growth factors also play a role in the control of bone cell activity.	FENG et al. (2011) Disorders of bone remodeling. https://doi.org/10.1146/annurev-pathol-011110-130203
Answer: Agree		
5. The bone is a dynamic tissue that undergoes constant physiological remodelling to maintain its structural integrity and mineral homeostasis.	The bone is in constant physiological remodelling to maintain structural integrity and mineral homeostasis.	BOYCE et al. (2012) The osteoclast, bone remodelling and treatment of metabolic bone disease. https://doi.org/10.1111/j.1365-2362.2012.02717.x; RAGGATT et al. (2010) Cellular and molecular mechanisms of bone remodeling.
Answer: Agree		
6. Bone loss in postmenopausal women is irreversible.	Hormone replacement, proper nutrition and regular physical activities are able to maintain bone homeostasis.	PASQUALINI et al. (2019) Effects of a 3-month weight-bearing and resistance exercise training on circulating osteogenic cells and bone formation markers in postmenopausal women with low bone mass. https://doi.org/10.1007/s00198-019-04908-9
Answer: Disagree		
7. In order for the orthodontic treatment to be successful in women, it is of the utmost importance that the orthodontist is aware of the hormonal effects during the various stages of female life.	It is necessary that the health professionals gather knowledge about the systemic condition of their patients so that the best diagnosis and prognosis for each specific situation can be established.	VILELLA et al. (2016) The Association of Oral Health Literacy and Oral Health Knowledge with Social Determinants in Pregnant Brazilian Women. https://doi.org/10.1007/s10900-016-0186-6
Answer: Agree		
8. A special orthodontic care protocol is required for pregnant, lactating and postmenopausal women.	It is important that the care provided is in accordance with the need and systemic condition of each patient at their time to undergo treatment. That assistance takes place in an individualized, personalized, and integral way.	GOVINDASAMY et al. (2018) Knowledge, awareness, and practice among gynecologists, medical practitioners and dentists in Madurai regarding association between periodontitis and pregnancy outcomes. https://doi.org/10.4103/jisp.jisp_164_18; LEE et al. (2010) Dentists’ perceptions of barriers to providing dental care to pregnant women. Womens Health Issues. https://doi.org/10.1016/j.whi.2010.05.007; STEWART et al. (2012) Building osteoporosis prevention into dental practice; VILELLA et al. (2016) The Association of Oral Health Literacy and Oral Health Knowledge with Social Determinants in Pregnant Brazilian Women. https://doi.org/10.1007/s10900-016-0186-6
Answer: Agree		

The questionnaire’s reliability was assessed through internal consistency and test-retest reliability. Cronbach’s alpha was 0.77 and McDonald’s omega was 0.78, demonstrating the good internal consistency of the questionnaire. In the test-retest, the ICC was 0.71, whereas Spearman’s correlation coefficient was 0.51, confirming the good test-retest reliability. Significantly higher average scores were observed in the second application of the questionnaire compared to the scores obtained in the first application (p = 0.01).

## Discussion

To the best of our knowledge, this study represents the first approach to the development and validation of an instrument to assess the level of knowledge of orthodontists during the treatment of pregnant, lactating, and postmenopausal women. Validity and reliability are key elements in the evaluation of a measurement instrument.^19^ In this sense, the main result of this study was the development of a valid and reliable instrument to assess the level of knowledge of orthodontists during the treatment of pregnant, lactating, and postmenopausal women.

There are different instruments in the literature to measure the delivery of health services to the general population.^20^ Previous studies have indicated the need to raise awareness among general dentists of oral health control in pregnant women,^21^ in addition to the need for measures to prevent osteoporosis in the practice of dentistry.^22^ However, few studies have assessed other aspects of orthodontic treatment, such as the care of pregnant, lactating, and postmenopausal women during oral health treatment.^2,22,23^ Considering this gap, we verified the need to develop a construct that would assess more specific parameters of the level of knowledge of orthodontists about the care of women in these specific stages of their lives.

A measurement instrument with good methodological quality is essential for the development of any scientific activity.^15,24^ The first step in the development of this instrument was to carry out a bibliographic survey, looking for a better definition of instrument development and validation, followed by the establishment of a focus group of experts with experience in the subject of this study. In this sense, validated and widely used protocols were followed in the development and validation of instruments.^13-16^ Correctly defining the target population to be studied, having a clear and objective language for the target population of the instrument,^19,24,25^ and building up the correct methodology for the good development of the study are important and necessary aspects during the process of development and validation of a construct.^15,16^ The definition of the studied population and the validation of this construct were obtained through a review of the literature, meetings with the group of experts, and access to the pilot groups’ suggestions for changes in the instruments.

Other important factors to address the development and validation of a construct are item generation, item reduction, questionnaire design, and questionnaire validation and reliability.^13-16^ Item generation, carried out by the group of experts, resulted in 60 items with five response options. Item reduction and questionnaire design have to be performed to assess the relevance, objectivity, understanding, and reduction of the number of items.^26-28^ In line with the literature, two pilot tests were run at different time points and with different purposes in the present study. The first pilot test was performed to assess the level of relevance of each item of the instrument presented in the item reduction phase. A second pilot test was used to verify the objectivity of the proposed questions to determine the questionnaire design after the analysis by the group of experts. In this study, each item of the developed instrument was duly substantiated by the literature, with at least one reference on the topic addressed by the item for content validity.

The final version of the questionnaire was applied to a sample of orthodontists to assess its validity and reliability. A construct is considered valid when it is capable of measuring what is intended to be measured.^20,26,29^ In this study, the questionnaire was evaluated for face and content validity. Face and content validity tests are meant to verify whether what is being measured with the selected items is really being measured.^30^ This validity is not determined by statistical analysis, but rather by a qualitative assessment carried out by the group of experts and orthodontists. The evaluations made by both groups showed that the instrument contains clear and objective questions and that the content and answer options are appropriate for what was being evaluated.

Reliability is related to the ability to measure the construct in a consistent, accurate, and stable manner.^16,24^ Internal consistency is the most widely used indicator of test reliability. In this study, the developed instrument presented satisfactory reliability, which was confirmed by Cronbach’s alpha test (0.77) and by McDonald’s omega test (0.78).^26,31^ Although the literature argues that Cronbach’s alpha values greater than 0.70 are considered appropriate, alpha values may be lower for scales with few items and in research in which the sample is homogeneous.^32^ On the other hand, McDonald’s omega coefficient is based on a similar model, demonstrating relevant values when this cutoff point is considered.^33^

Most of the studies test-retest the instrument in an average time of 15–30 days.^14,26,27^ In this study, the mean application period of the test-retest had a mean of 45 days after the test application. One possible explanation for this delay to obtain the answers was probably due to the fact that the questionnaire was developed, tested, and test-retested during the COVID-19 pandemic. The reliability of a construct tends to decrease when the test-retest application is extended.^34^ This survey was reapplied with a mean of 45 days, showing a low Spearman’s correlation coefficient. Regardless of this fact, our instrument was developed to analyze the knowledge of the orthodontist and there is no consensus in the literature about the test-retest interval for this study profile. Since the instrument developed in this study was an assessment of knowledge rather than of the participant’s current health status, such as in the Parental-Caregiver Perceptions Questionnaire (P-CPQ), the slightly longer interval does not affect the current data. Interestingly, in this study, important differences in scores were found between the first and second questionnaire applications. In this sense, participants may have been interested in looking for the right answers after answering the questionnaire. Corroborating this hypothesis, significantly higher average scores of correct answers were observed in the second application of the questionnaire. Anyway, the questionnaire showed good test-retest reliability, demonstrating the instrument’s stability. Certainly, in this study, the interval between applications was large, requiring a test-retest with a shorter interval between applications in the future.

According to the proposed sample size calculation method,^14^ the sample size for a validation study should be 5 to 7 times greater than the number of items in the instrument, requiring at least 100 participants. The number of participants was determined according to the proposed sample calculation method. Thus, the final number of participants included in this study was 258. The present study included orthodontists from several Brazilian states, thereby improving the reliability of the results.

Although the present paper showed satisfactory results, further studies are needed to assess the structural validity of the instrument and also to evaluate the psychometric properties in distinct populations. The main features of this instrument should be considered for future improvements or as a basis for developing new instruments that aim to assess levels of knowledge in different dental specialties.

Despite reports in the literature on the concern of orthodontists with oral hygiene during orthodontic treatment,^35^ few studies have addressed other aspects of orthodontic treatment such as the care of pregnant, lactating, and post-menopausal women.^2,3,4,23^ A recent study has shown that most patients who seek and accept orthodontic treatment are women.^36^ This raises the concern about whether orthodontists are able to treat these women in their different stages of life, making it necessary to create a questionnaire to assess the orthodontists’ knowledge. To date, there has been no instrument addressing this issue, thus increasing the relevance of the present study. This instrument can be widely used in Brazil, as there are 30,093 registered orthodontists, making orthodontics the dental specialty with the highest number of professionals in the country. Also, the questionnaire will provide access to the knowledge of these specialists about these patients and their treatment.

## Conclusion

The instrument “Considerations on Orthodontic Treatment during Pregnancy, Lactation, and Postmenopausal periods” was developed and validated. This instrument proved to have satisfactory applicability, validity, and reliability to assess the level of knowledge of orthodontists about the orthodontic treatment of women in their different stages of life. This instrument can be used to encourage orthodontists to seek constant learning about the topic and also encourage individualized, personalized, and comprehensive orthodontic practice concerning pregnant, lactating, and postmenopausal women. Therefore, its use in the field of orthodontics is promising.
